# Effects of Fiber Density and Strain Rate on the Mechanical Properties of Electrospun Polycaprolactone Nanofiber Mats

**DOI:** 10.3389/fchem.2020.00610

**Published:** 2020-07-21

**Authors:** Adriano A. Conte, Katie Sun, Xiao Hu, Vince Z. Beachley

**Affiliations:** ^1^Department of Biomedical Engineering, Rowan University, Glassboro, NJ, United States; ^2^Department of Materials Science and Engineering, Rutgers University, New Brunswick, NJ, United States; ^3^Department of Physics and Astronomy, Rowan University, Glassboro, NJ, United States

**Keywords:** nanomaterials, material testing, mechanical properties, electrospinning, polymer

## Abstract

This study examines the effects of electrospun polycaprolactone (PCL) fiber density and strain rate on nanofiber mat mechanical properties. An automated track collection system was employed to control fiber number per mat and promote uniform individual fiber properties regardless of the duration of collection. Fiber density is correlated to the mechanical properties of the nanofiber mats. Young's modulus was reduced as fiber density increased, from 14,901 MPa for samples electrospun for 30 s (717 fibers +/– 345) to 3,615 MPa for samples electrospun for 40 min (8,310 fibers +/– 1,904). Ultimate tensile strength (UTS) increased with increasing fiber density, where samples electrospun for 30 s resulted in a UTS of 594 MPa while samples electrospun for 40 min demonstrated a UTS of 1,250 MPa. An average toughness of 0.239 GJ/m^3^ was seen in the 30 s group, whereas a toughness of 0.515 GJ/m^3^ was observed at 40 min. The ultimate tensile strain for samples electrospun for 30 s was observed to be 0.39 and 0.48 for samples electrospun for 40 min. The relationships between UTS, Young's modulus, toughness, and ultimate tensile strain with increasing fiber density are the result of fiber-fiber interactions which leads to network mesh interactions.

## Introduction

Polymer nanofibers with diameters from tens of nanometers to a few micrometers can be fabricated using the electrospinning method. Unique physical nanoscale effects occur in materials with dimensions <100 nm, but fibers with diameters from 1 to 999 nanometers are commonly referred to as nanofibers in the electrospinning literature due their diameters residing in the nano order of magnitude. Advantageous features of electrospun nanofibrous materials, as a result of their high surface area to volume ratio, include exceptional strength per unit mass, high surface energy, and ability as a barrier to prevent liquid penetration. An extensive array of applications may make use of these valuable traits for future generations of fabricated materials (Patanaik et al., 2007). Research has been performed for the development of lightweight protective attire with electrospun materials in addition to other applications such as tissue replacement grafts and drugs carriers (Gibson et al., 2001; Kenawy et al., 2002; Schreuder-Gibson et al., 2002; Katti et al., 2004; Schreuder-Gibson and Gibson, 2006; Zhang et al., 2006; Lee and Obendorf, 2007a; Beason et al., 2012; Mun et al., 2012; Garrigues et al., 2014). As barrier materials, thin nanofiber mats exhibit high tensile strength, flexibility, and reduced permeability of water and air with increasing mat density (Lee and Obendorf, 2007b). Electrospinning allows exceptional versatility to produce fiber mats with targeted properties. Altering electrospinning parameters and collection methods can enhance fiber molecular orientation, which has been observed to result in enhanced mechanical properties (Jiang et al., 2018a; Yang et al., 2018). Various additives can be directly doped into electrospinning solutions to tune performance as well. One study varied the amount of diphehyl phosphate in polyimide fibers from 0 to 0.9 wt% at intervals of 0.2 wt%. In that study it was found that strength systematically increased from 23 to 32 MPa in the 0 to 0.9 wt% groups, as well as modulus from 0.7 to 1.5 GPa, and finally toughness from 3.8 to 6.9 MPa (Wang et al., 2020). These approaches in conjunction with varying the overall fiber density may allow for the production of high performance fiber mats tailored to specific applications. Low fiber density nanofiber films are well-suited to several applications. For example, they can be stacked to form high efficiency, low resistance filters, and are ideal for aligned tissue scaffold constructs. Ultra-thin nanofiber arrays with optimized fiber density demonstrated the capacity to produce highly cellularized aligned tissue sheets with an extremely low biomaterial to cell volume ratio (Beachley et al., 2014). These low density ultra-thin materials have the potential to suit applications in tissue engineering including wound healing and muscle regeneration (Beachley and Wen, 2009).

The current study seeks to experimentally investigate the relationship between fiber density and mechanics for electrospun aligned nanofiber thin films. While the mechanical properties of ideal isotropic materials are constant among varying sample geometries and sizes, this does not hold true for fibrous mats and other materials that do not maintain consistent properties irrespective of size or geometry (Adham et al., 1996; Matthews et al., 2002; Zong et al., 2003; Shin et al., 2006; Sun et al., 2006; Kai et al., 2011; Sperling et al., 2017). It is crucial to understand how mechanics scale with fiber mat density in an effort to make accurate comparisons of measured nanofiber structure mechanics between studies, as well as laying the foundation for the possibility of predictive scalability of various sized fibrous constructs. This idea is important to keep in mind when specifically characterizing scaffolds of various geometries and sizes for a particular application.

Previous work has shown that electrospun mat thickness is a determinant of the mechanical properties of the final structures. Packing density of an electrospun material has been shown to play a large role on the properties of electrospun materials in comparison to mat thickness (Leung et al., 2010). One study observed the effects of electrospun polycaprolactone fiber mat thickness on indentation modulus, and found that an increase in mat thickness resulted in an increase in indentation modulus in supported mats (Calhoun et al., 2019). Additionally, a 1.5x increase in Young's modulus was observed in unsupported polycaprolactone mats when comparing a 50 um thick mat to a 200 um thick mat. Tensile strength has been shown to more than double as the thickness of polycaprolactone mats was increased from 0.02 mm (5.6 MPa) to 0.03 mm (12.8 MPa) at a testing speed of 50 mm/min (Doustgani et al., 2012). However, this increase in tensile strength has not been observed across the board, as was the case in poly(d,l-lactide) and poly(l-lactide) where there was an observed reduction in tensile strength (1.153 to 0.676 MPa) in mats with thicknesses of 0.2 and 0.25 mm, respectively (Wright et al., 2010). Reported mechanical properties for single electrospun fibers are commonly much higher compared to fiber meshes (Jiang et al., 2004; Tan et al., 2005; Li et al., 2006; Lim et al., 2008; Wong et al., 2008; Zhang et al., 2009; Ladd et al., 2011; Chen et al., 2013; Pauly et al., 2016; Brennan et al., 2018). Incredibly high tensile strengths of 1,000–2,500 MPa have been observed specifically for single electrospun polyimide fibers from electrostatically and thermally induced molecular orientation in the direction of the fiber axis (Chen F. et al., 2008; Chen et al., 2012, 2015; Xu et al., 2017). Yet even more single fiber studies have reported tensile strength values of 40–3,500 MPa, and Young's modulus values anywhere between 0.36 and 502 GPa (Jiang et al., 2018b). However, it may be difficult to directly compare these studies since the electrospinning conditions, mechanical testing methods, and number of fibers in each structure are different across studies. For example, single fiber samples are commonly collected using the parallel plate method, which may produce stronger fibers, although cross-sectional areas of thicker meshes measured with a caliper may not be able to accurately account for pore spacing when calculating cross-sectional area. The literature clearly shows that fiber density will affect the mechanical properties of collecting electrospun nanofiber meshes, however highly controlled investigations of these relationships are limited.

The current investigation seeks to expand upon the cumulative mechanical property effects of electrospun fiber density, particularly ultra-thin low density mats, which have seldomly been studied. Experiments were conducted by means of the versatile candidate polymer polycaprolactone (PCL) utilizing an automated track collection system. PCL is a synthetic polymer that has been regularly used for medical applications as it exhibits slow biodegradation and biocompatible characteristics (Venugopal et al., 2005; Agarwal et al., 2008; Neppalli et al., 2010; Van der Schueren et al., 2011). Furthermore, PCL has been widely used in electrospinning studies due to its ease of use and compatibility with a multitude of electrospinning collection configurations. An automated track system uniquely allows for precise control over the fiber number per mat in aligned nanofiber arrays and maintains a consistent electrical field at the collection area irrespective of final mat fiber density to limit the possibility of macromolecular variations between fibers in mats of various densities (Conte et al., 2019). The end to end tension on collected fibers and the consistency of the electrical field are critical to limiting processing artifacts associated with polymer chain relaxation after collection and collector charge repulsion effects respectively (Reneker et al., 2000; Theron et al., 2004; Greiner and Wendorff, 2007; Beachley et al., 2013; Ma et al., 2016). Ultra-thin aligned nanofiber films with precise nanofiber density could be suitable for applications such as lightweight attire, tissue engineering replacement grafts, and filtration. In the field of tissue engineering, large area ultra-thin nanofiber sheets with tunable fiber orientation and density could possibly serve the application of producing confluent cell sheets with minimal interference of cell-cell contacts, adequate surface area cell attachment, and mass transport (Chen M. C. et al., 2008; Beachley et al., 2014). Understanding the relationship between fiber density and mechanics in thin polymer nanofiber films is valuable to engineering thin nanofiber film devices and thin film composite structures.

## Materials and Methods

### Automated Track Electrospinning System

**Figure 1:**
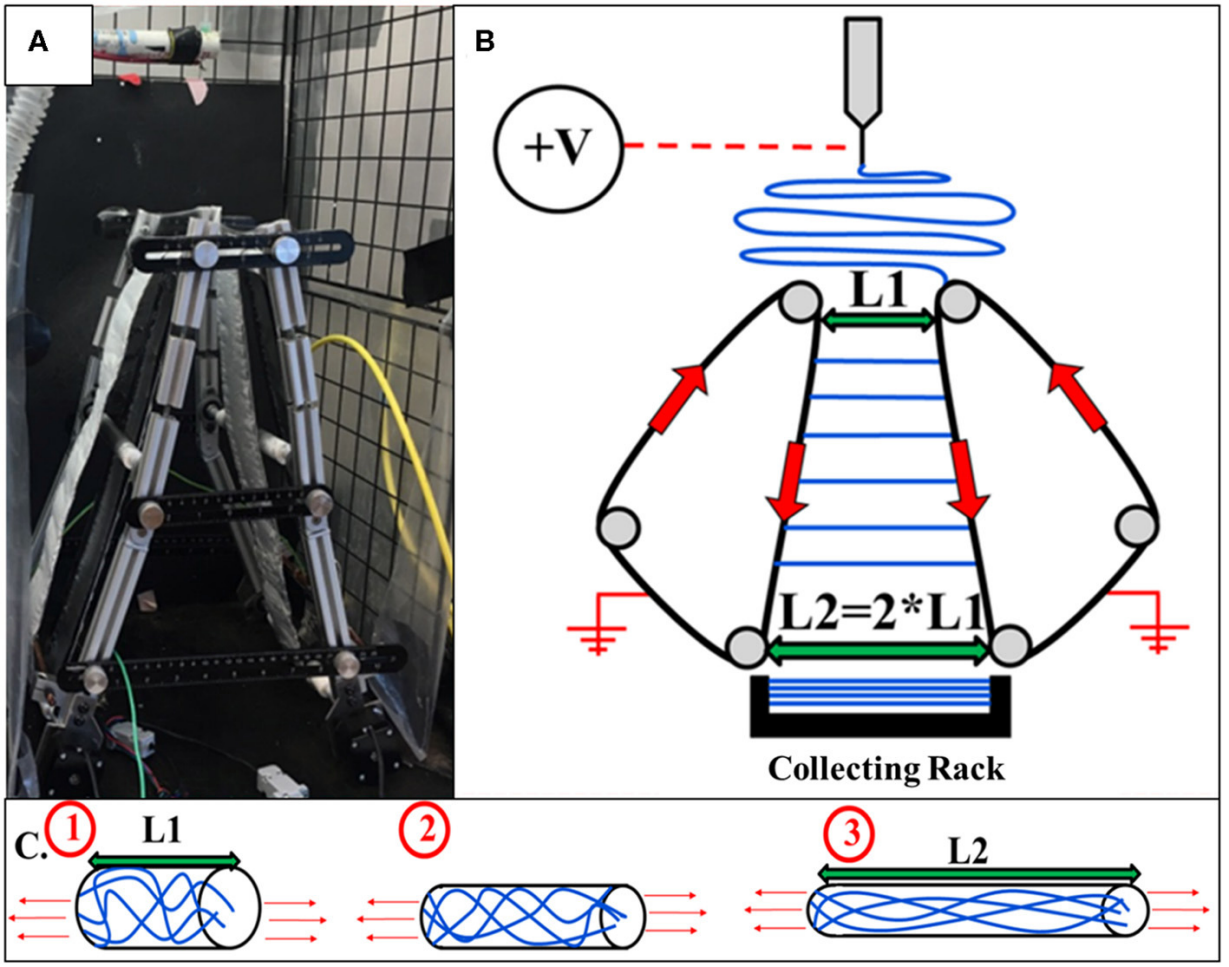
**(A)** Automated adjustable track system capable and collecting and post-drawing thousands of electrospun nanofibers individually. **(B)** A diagram illustrating the collection and extension of electrospun nanofibers using the revolving automated tracks. **(C)** A representation of the macromolecular structure of a polymer chain at the top of the automated tracks (1), in the middle of the tracks (2), and at the bottom of the tracks as well as in the collecting rack (3).

An automated parallel track system with adjustable track angles was implemented to collect and post-draw electrospun polycaprolactone (PCL) nanofibers. The automated track system was constructed for the purpose of limiting residual charge accumulation effects and polymer chain relaxation. Angled tracks make it possible to post-draw individual nanofibers during collection. The frame was constructed of aluminum t-slots. For track fabrication, 0.007 inch aluminum tape was wrapped around the rotatable rods connecting the front and rear frames of the system at the top and bottom of the device. The aluminum parallel tracks were rotated utilizing two Nema stepper motors which turn the bottom rods. Figure 1 displays the automated track system utilized for the current study as well as a diagram.

### Electrospinning

18 wt% PCL of a molecular number of Mn = 80,000 (Sigma Aldrich) was dissolved in a 3:1 solvent system of dichloromethane (DCM) and dimethylformamide (DMF) in 20 mL vials. The solutions were mixed overnight at room temperature. Electrospinning was carried out at 10 kV with a needle height of 17 cm between the tip of the needle to the collection tracks of a custom collector system (Brennan et al., 2016). To assess the effects of electrospun nanofiber density, electrospun samples were produced in separate trials for intervals of 30 s, 3 min, 10 min, 15 min, 25 min, and 40 min. Samples were electrospun and collected at a maximum of 40 min, beyond this point samples became too thick to accurately quantify fiber counts and diameters. The maximum duration was selected at 40 min due to the inability to accurately assess fiber density from scanning electron microscopy (SEM) images beyond that time point. The syringe pump (New Era Pump Systems) ejected the PCL polymer solution at a flow rate of 0.8 mL/h through a 21 gauge needle. The electrospinning apparatus was contained within an acrylic chamber to maintain desired humidity (45–55% RH) conditions. Electrospun samples were post-drawn to a length twice their original length at draw ratio 2 (DR2), where the initial top gap between tracks was positioned at 4 cm apart, and the final bottom gap between parallel tracks was set to 8 cm apart. All samples were fixed to plastic window frames with square openings of 10 × 10 mm. Six individual sample mats were produced and tested for each electrospinning duration (*n* = 6). Each of the six samples of each duration were taken from its own individual collection tray after electrospinning, and adhered to the above mentioned window frames.

### Imaging

Fiber cross-sectional area and density were ascertained using SEM imaging (Phenom Pure Desktop SEM, Phenom, Netherlands). Five images at 850x magnification were taken for fiber density information as well as five images at 8,500x magnification for measuring fiber diameters. The Cell Counter feature in ImageJ software was utilized to enumerate the number of fibers within a distance of 100 um orthogonal to the fiber alignment direction. Fiber diameter was recorded using a measurement tool within the ImageJ software. The total number of fibers per sample was ascertained by taking the average number of fibers per 100 um for a given sample and multiplying that by 100 for a 100 mm total sample width. Average cross-sectional area was determined using the equation 0.25^*^π^*^(Average Diameter)^2^. Total cross-sectional area was obtained through multiplying average cross-sectional area for a given sample by the total number of fibers. The ImageJ plugin called Directionality was utilized to assess fiber orientation with respect to the horizontal axis of the image. Accurate highlighting of fibers was validated by manual inspection of the orientation map generated by the plugin. Surface area of the fiber samples was computed using the diameter of fibers, fiber counts, and length of the window frame (10 mm).

### Mechanical Testing

All mechanical testing and related analysis were performed on samples post-drawn to a draw ratio of 2 (DR2) which were produced by the automated track system mentioned in this study. Samples with 10 × 10 mm dimensions were mounted on plastic frames for mechanical testing. The frame mounted PCL fiber mats were tested in a Shimadzu EZ-SX (Shimadzu; Kyoto Japan) mechanical tester at strain rates of 0.5, 5, 50 mm/min. A sample size of six was used at each strain rate for each of the electrospinning durations. Stress was determined as the force values outputted by the Shimadzu mechanical tester divided by the average cross-sectional area value calculated using average fiber diameter and total fiber number (determined from SEM images). Engineering strain was calculated as the displacement outputted by the mechanical tester divided by the original length of the sample (10 mm). Young's modulus was determined from the slope of the linear region of stress strain curves at strains between 0 and 0.35. Toughness was determined by taking the area under the stress strain curves. During tensile testing, mechanical failure is assumed to be the point after which the force displacement curve reaches its maximum.

## Results

**Figure 2:**
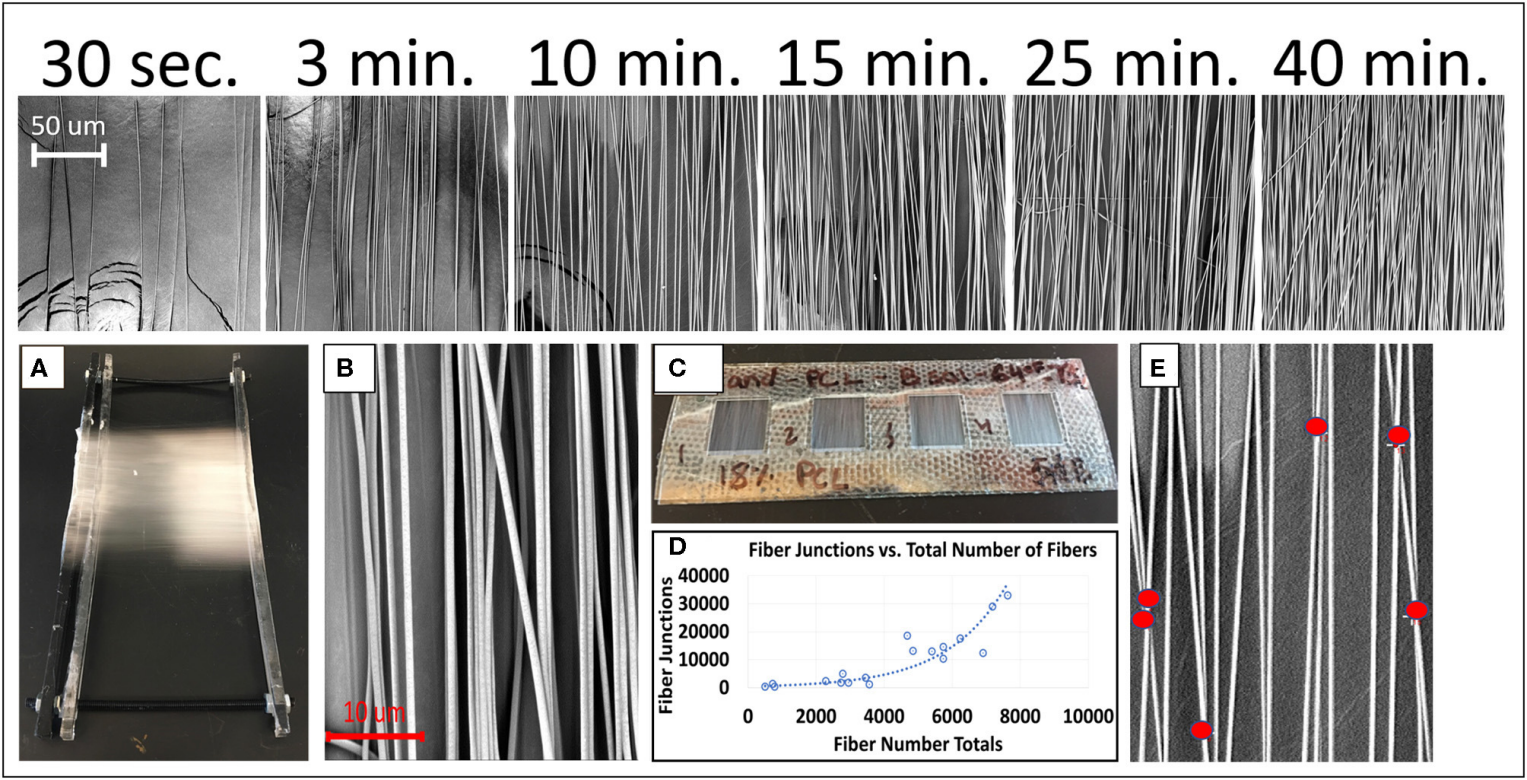
(Top) Representative electrospun PCL nanofibers by electrospinning duration captured at 1,500x magnification. **(A)** PCL nanofibers adhered to collection tray. **(B)** SEM image of electrospun fibers at 8,000x magnification specifically used for measuring fiber diameters. **(C)** Close up of 10 × 10 mm plastic window squares containing PCL nanofibers. **(D)** Graph depicting fiber junction counts to total number of fibers. **(E)** SEM image of electrospun PCL nanofibers captured at 8,000x magnification with red circles used to quantify fiber junctions.

Figure 2 depicts photographs and SEM images of PCL nanofibers by electrospinning duration. The fiber morphology is shown to be mostly aligned through collection with the automated parallel track system. It can be observed in Figure 2 (top), that as electrospinning duration increases there is also a relative increase in the number of fibers as expected. Figures 2A–C display the predominantly aligned electrospun PCL nanofibers adhered to the collection tray that is located under the automated track system during collection time. In addition, the window frames containing the PCL fibers and utilized for SEM imaging and tensile testing is displayed. Figure 2D presents an exponential relationship existing between total fiber junctions and total fibers. Exponential, linear, logarithmic, and power functions were all considered for representing the overall trend of the data, with an exponential function being implemented due to it being the highest *R*^2^ value in comparison to the other functions. Figure 2E depicts an example of how fiber junctions were quantified.

**Figure 3:**
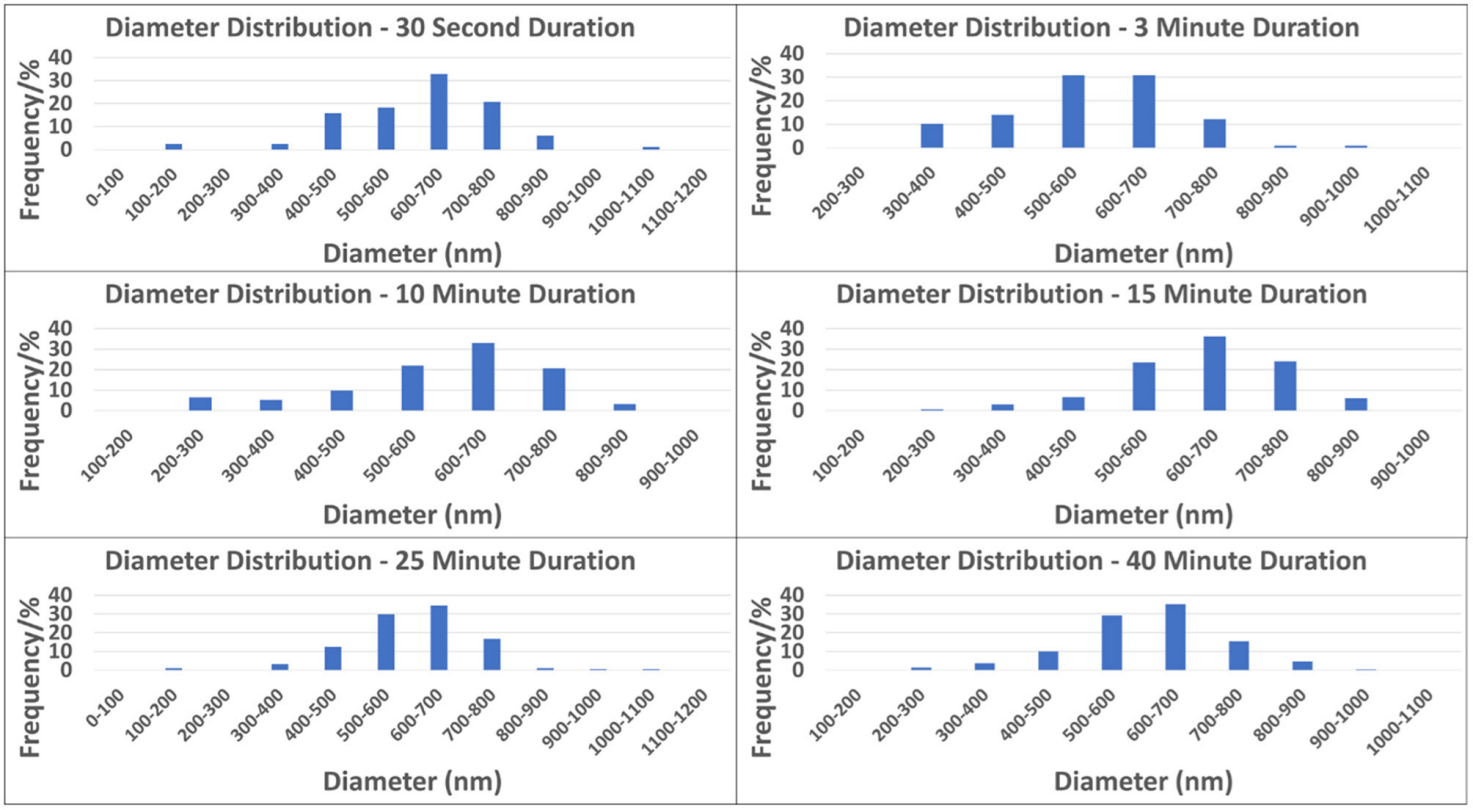
Diameter distributions by each of the electrospinning durations.

**Figure 4:**
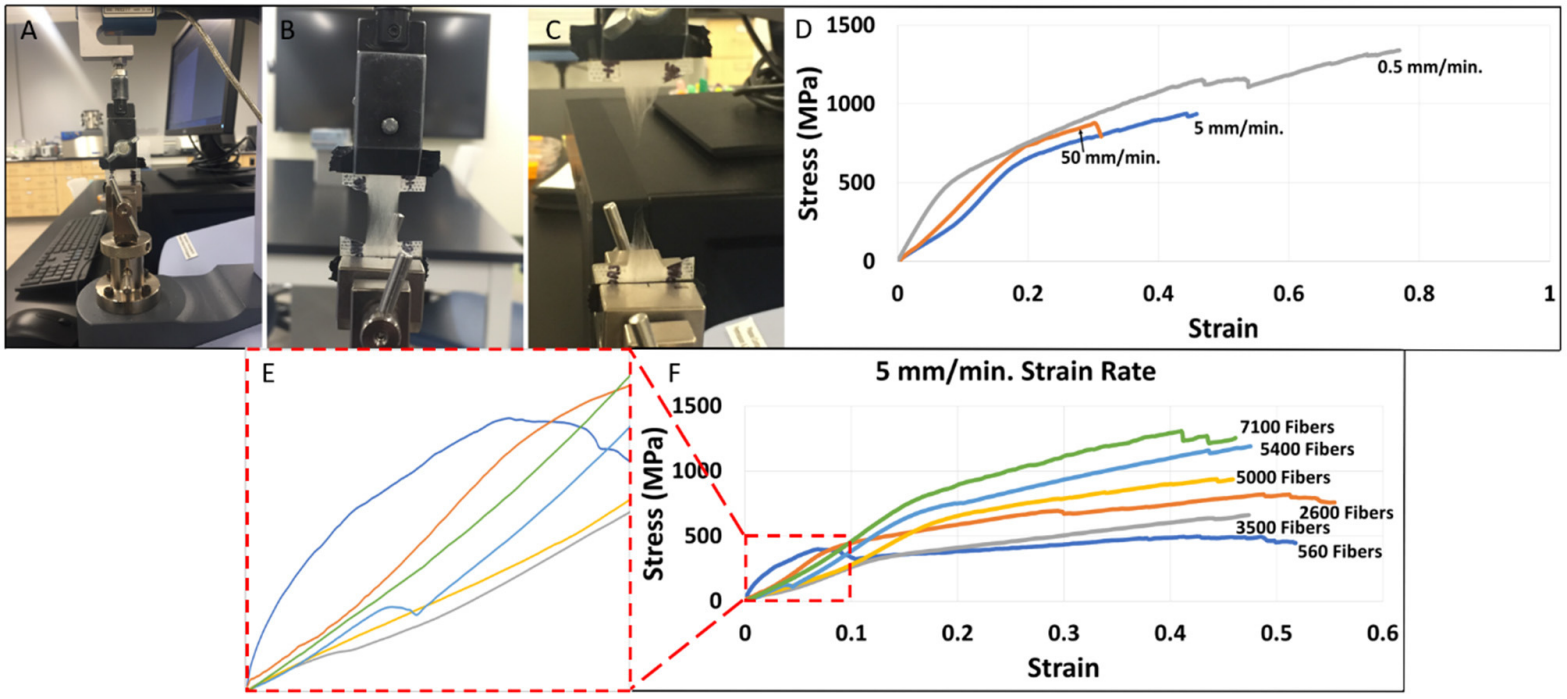
**(A)** Initial tensile testing setup performed on a Shimadzu EZ-SX mechanical tester. **(B)** Elongation of electrospun PCL nanofibers during testing. **(C)** Failure of electrospun PCL nanofibers at the conclusion of tensile testing. **(D)** Representative stress-strain curves by strain rate (mm/min.) for individual fiber mat samples containing 4,753–4,976 fibers. **(E)** Enlarged linear regions of the stress-strain curves by fiber number where Young's modulus values were selected. **(F)** Stress-strain curves by fiber number at a 5 mm/min. strain rate (individual representative samples).

Figure 3 depicts the distribution and frequency of fiber diameters at each electrospinning duration. Table 1 (top) specifically lists the average fiber diameter and standard deviation for each duration. In addition, Table 1 (middle) lists the average orientation in degrees of fibers with respect to the horizontal axis of the image for all durations. Both diameter and orientation remains statistically the same regardless of electrospinning duration. Table 1 (bottom) shows the average total surface area of samples increasing for each successive electrospinning duration. Figures 4A–C presents the overall process of tensile testing for the electrospun PCL nanofibers from the initial setup through to material failure. Figure 4D depicts representative stress-strain curves for one sample each at the 0.5, 5, and 50 mm/min. strain rates. The stress-strain curves in Figure 4F are presented by fiber number. It can be observed that the sample containing 560 fibers has an ultimate tensile strength (UTS) of <500 MPa, whereas the sample containing 5,000 fibers has a UTS at ~1,000 MPa. Furthermore, the sample containing 7,100 fibers exhibited a UTS of ~1,300 MPa. These observations demonstrate an overall trend of increased UTS with increasing total number of fibers.

**Table 1:** Average diameter and range by duration (top), average fiber orientation and standard deviation (middle), and average total surface area (nm^2^) for each sample duration.

**Diameter distribution**			
**Duration**	**Average diameter (nm)**	**Standard deviation**	**Range (nm)**
30 s	614.21	134.72	185–1,062
3 min	571.40	118.36	326–900
10 min	592.48	140.43	235–842
15 min	626.17	111.97	282–862
25 min	593.01	118.17	142–1,111
40 min	604.04	117.16	279–903
All durations	600.22	123.47	142–1,111
**Fiber orientation**			
**Duration**	**Average direction with respect to horizontal axis of image (°)**	**Average dispersion (°)**—**standard deviation**	
30 s	87.57	6.12	
3 min	89.40	4.57	
10 min	88.26	4.20	
15 min	89.26	4.45	
25 min	87.19	3.25	
40 min	88.18	3.42	
All durations	88.30	4.28	
**Surface area**			
**Duration**		**Average total surface area (nm**^**2**^**)**	
30 s		1.41E + 13	
3 min		3.36E + 13	
10 min		7.22E + 13	
15 min		1.00E + 14	
25 min		1.34E + 14	
40 min		1.57E + 14	
All durations		8.50E + 13	

**Figure 5:**
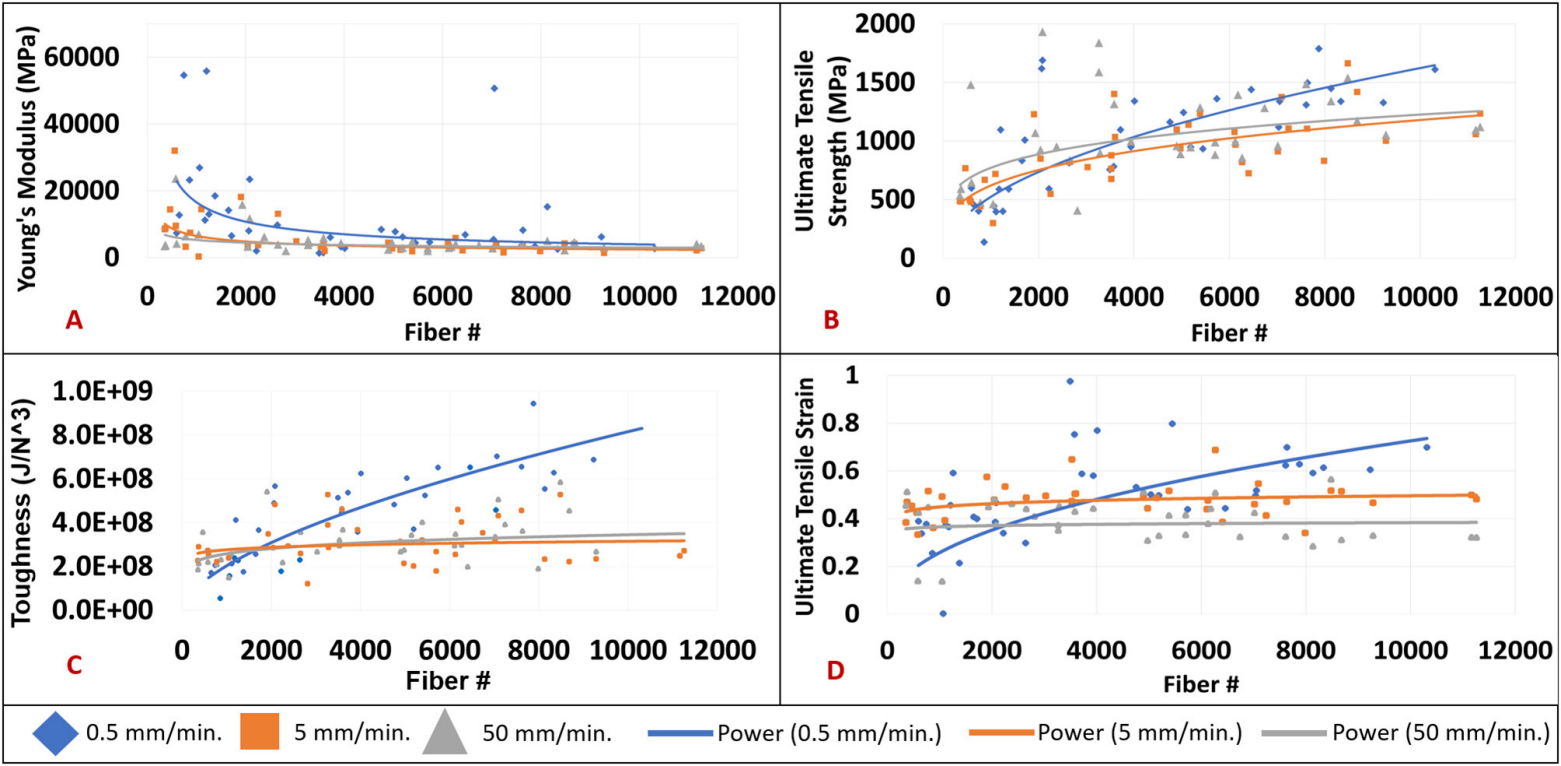
**(A)** Young's modulus (MPa) by fiber number at strain rates of 0.5 mm/min. (blue diamond), 5 mm/min. (orange square), and 50 mm/min. (gray triangle). **(B)** Ultimate tensile strength (MPa) by fiber number at strain rates of 0.5 mm/min. (blue diamond), 5 mm/min. (orange square), and 50 mm/min. (gray triangle). **(C)** Toughness (J/N^3^) by fiber number at strain rates of 0.5 mm/min. (blue diamond), 5 mm/min. (orange square), and 50 mm/min. (gray triangle). **(D)** Ultimate tensile strain by fiber number at strain rates of 0.5 mm/min. (blue diamond), 5 mm/min. (orange square), and 50 mm/min. (gray triangle).

Figure 5 compares the mechanical properties of nanofiber mats with different fiber densities tensile tested at different strain rates. A power function trendline was drawn on all of the graphs for visualization purposes. The power function was selected due to the highest *R*^2^ value in comparison to exponential, linear, logarithmic, and polynomial functions. Figure 5A shows an inverse relationship between Young's modulus and increasing fiber number that is most pronounced at a strain rate of 0.5 mm/min. In observing the data, a large reduction in Young's modulus can be seen particularly when fiber samples consist of <2,000 nanofibers. An overall reduction in Young's modulus is observed in increasing strain rate groups of 0.5 to 50 mm/min. The Young's modulus values demonstrated an overall reduction from an average of 14,901 MPa for samples electrospun at 30 s (717 fibers +/– 345) to 3,615 MPa in the samples electrospun at 40 min (8,310 fibers +/– 1,904). The percent change would amount to a 76% reduction in Young's modulus values from the 30 s group to the 40 min group. Figure 4B demonstrates an increase in ultimate tensile strength (UTS) with increasing fiber number for all strain rates. The increase in UTS is shown to be more substantial for increasing fiber number than it is for increasing strain rate. The UTS at 30 s samples was observed to be at 594 MPa while samples electrospun for 40 min demonstrated average UTS values of 1,250 MPa. The overall increase would amount to 110% from the 30 s samples to the 40 min samples.

Figure 5C shows an increase in toughness with increasing fiber number. Furthermore, there is a strong positive correlation between increased toughness and the 0.5 mm/min. strain rate. However, there is no relationship between toughness and strain rates of 5 or 50 mm/min. irrespective of fiber number. An average toughness of 0.239 GJ/m^3^ was observed in the 30 s group, while a toughness of 0.515 GJ/m^3^ was seen in the 40 min group. This accounts for 113% increase in overall toughness. Figure 4D demonstrates an increase in ultimate tensile strain with increasing fiber number at the 0.5 mm/min strain rate. There is minimal change observed between ultimate tensile strain and the 5 or 50 mm/min strain rates regardless of fiber number. The average ultimate tensile strain for the 30 s group was observed to be at 0.39, and 0.48 in the 40 min group which accounts for a 22% increase. Scatter in the data observed in Figure 4 could be attributed to error in measurements of the cross-sectional area based on standard deviations of fiber diameters and fibers counted across images or experimental error associated with frame mounting procedure and frame trimming prior to testing. No outliers were removed in the presented data.

## Discussion

In contrast to the current work's mechanical results, several other studies of aligned electrospun PCL mats have demonstrated much lower ultimate strength ranges of 1.4–6.9 MPa and Young's moduli values of 3.3–75 MPa (Jiang et al., 2004; Kai et al., 2011; Chen et al., 2013; Pauly et al., 2016). The UTS values of those studies are three orders of magnitude lower than the samples electrospun for 40 min in this study (1,250 MPa) and two orders of magnitude lower for Young's moduli values observed also at 40 min (3,615 MPa). Furthermore, studies on randomly oriented electrospun PCL have shown even lower ultimate tensile strength values of 1–2.38 MPa and Young's moduli values of 8.5–31 MPa (Jiang et al., 2004; Li et al., 2006; Zhang et al., 2009; Kai et al., 2011; Ladd et al., 2011; Chen et al., 2013; Pauly et al., 2016). Although the current study far exceeds the prior mentioned studies in UTS values, there has been other studies in the literature where both conventionally post-drawn PCL microfibers and electrospun PCL nanofibers have had similar high values of UTS (200–800 MPa) (Mochizuki et al., 1995; Lim et al., 2008). The Lim et al. study used a parallel plate collection system which aids in the electrostatic inducement of aligned fibers acrossto the plates both at a macro and molecular level. The parallel plate configuration is very similar to the current study's parallel automated track system in that an electric field stretches nanofibers across a gap and may induce high macromolecular alignment. Also, while Lim et al. tested fibers individually, similarly the current study imaged individual fibers and diameter within mats to obtain a cross-sectional area that does not count pore space for the stress value calculations. These methodologies resulted in similar UTS values due to the inclusion of single fiber properties. There may be substantial error that has existed in numerous electrospun fiber studies wherein the quantification of cross-sectional area, and ultimately the assessment of fiber mechanical properties could be inaccurate due to the lack of accounting for spaces between fibers within a given fiber mat. Rather than utilizing a micrometer for measuring mat thickness, the present study only quantifies fiber cross-sectional area through SEM imaging, individual fiber counting, and fiber diameter measurements.

With regards to strain rate, a study observing the effects of strain rate on PAN fiber mats found higher ultimate tensile strength (130 MPa) and ultimate tensile strain were the result of lower strain rates (Naraghi et al., 2007). The previously mentioned study follows a similar trend where the current study demonstrated increased tensile strength and strain values at lower strain rates of 0.5 mm/min as can be observed in Figure 5.

Several studies have specifically investigated the effect of nanofiber mat thickness on the mechanical properties. Increasing electrospun mesh thickness for both random and aligned PCL mats resulted in reductions in peak stress (35–5 MPa), toughness (9–2 MPa), and Young's modulus at 0.1–0.3 strain with increasing mat thickness from 18.64 to 75.63 μm (Mubyana et al., 2016). The relationship is in contrast to the current study, which observed increases in ultimate tensile strength and toughness as fiber number per mat was increased (Figure 5). The previous study specifically tested thin (18.64 um), medium (52.18 um), and thick (75.63 um) aligned mats. It's important to note that the current and aforementioned studies exhibited an almost constant failure strain for all sample densities. Furthermore, any enhancement in mechanical properties observed for both ultimate tensile strength and toughness could be due to fiber-fiber interactions and the resulting friction or abrasion that they produce. Another study found that electrospun PCL scaffolds with a densely packed cross-section demonstrated increases in ultimate tensile stress (0.5 MPa loose to 0.9 MPa dense), ultimate tensile strain (0.55 loose to 1 dense), and Young's modulus (0.5 MPa loose to 1 MPa dense) in comparison to electrospun PCL scaffolds with a loosely packed cross-section (Soliman et al., 2011). The previously mentioned study modulated packing density of their samples and thus their sample fiber number through varying the distance of the capillary needle to the collector in addition to implementing a copper ring just below the needle. Sample thicknesses were kept consistent at 50–60 μm. That study follows the same trend as the current study's data in that more densely packed mats may have had increased friction at fiber-fiber junctions, which may ultimately play a larger role in overall mesh mechanics. These increases in mechanical properties were said to be proportional to reductions in pore diameters observed in densely packed fibers in comparison with sparsely packed fiber samples. In the aforementioned study, denser fiber mats behaved as multilayered composites that failed through delamination due to mode II cracking where in contrast the less dense specimens showed the same characteristics as fiber bundles that fail by mode I cracking. Mode I fracture or opening mode occurs when tensile stress exists perpendicular to the plane of the fracture. Mode II fracture or sliding mode occurs when a shear stress is acting parallel to the plane of the fracture as well as perpendicular to the fracture front (Barsom and Rolfe, 1999; Campbell, 2012; Sun and Jin, 2012). Based on the changes in mechanical properties that coincide with the exponential increase in fiber junctions with increasing fiber number, the current study may provide support for an influence of fiber-fiber interactions on the mechanics of multifiber meshes in thin, low fiber density meshes.

With respect to processing artifacts, it has been demonstrated that charged fiber accumulation that occurs during electrospinning alters the electric field, and consequently reduces the electrostatic forces that induce macromolecular alignment of fibers across a particular collection target (Ma et al., 2016). The reduced electrostatic forces due to fiber accumulation resulted in reduced macroscopic alignment of those fibers collected at the end of the time duration of collection (Ma et al., 2016). The electrostatic force across a parallel plate target also effects the macromolecular alignment of collected fibers, thus changes in the electrostatic force due to charge repulsion could cause macromolecular variation of deposited fibers.

Due to this phenomena that exists in the electrospinning process, the last fiber collected may have a different macromolecular arrangement than the first. In response to this dilemma, the automated track collection system, which constantly moves charged fibers away from the deposition area, should retain a uniform electrical field to mitigate the charge repulsion and electric field alteration concerns typically associated with denser electrospun mesh collection. Thus, we expect that this collection system is capable of producing electrospun meshes with limited macromolecular variation between fibers collected at the beginning and end of the collection period: a critical condition of this study.

Based on the values from Table 1 the current study finds that the macroscopic orientation of fibers is not statistically different from one electrospinning duration to another. We hypothesize that this is a result of the automated track system which maintained a constant electrical force at the deposition area throughout the duration of collection. It is also of note in Table 1 that the diameter of these fibers from group to group are also not statistically different, which also provides evidence that individual fibers from one duration group to the next are unchanged. Both of these results support our hypothesis that the electrostatic force on fibers in each sample are uniform and only fiber number is changed.

## Conclusion

The exponential relationship between fiber junctions and total fiber counts may have resulted in relationships between fiber mat mechanical properties and fiber density. Increasing electrospun PCL fiber density resulted in substantial increases in ultimate tensile strength at all strain rates. In contrast, a reduction in Young's modulus occurred at all strain rates, but particularly at the 0.5 mm/min strain rate. A reduction in Young's modulus may have been the result of shorter fiber segments between fiber-fiber junctions which impedes the ability of the fibers to re-organize in alignment with increasing strain. An increase in toughness and ultimate tensile strain was demonstrated at the 0.5 mm/min strain rate while small changes were observed in the 5 and 50 mm/min strain rates. The mechanical property enhancement observed in denser samples for ultimate tensile strength, toughness, and ultimate tensile strain may be attributed to increased fiber packing density in addition to fiber to fiber interactions. Lastly, an automated track collection system allows for consistent electrostatic force on fibers at the deposition area to promote uniformity of fibers at all collection durations/sample fiber densities.

## Data Availability Statement

The raw data supporting the conclusions of this article will be made available by the authors, without undue reservation.

## Author Contributions

KS and VB: conceptualization and methodology. AC and KS: formal analysis, investigation, and data curation. VB and XH: resources and funding acquisition. AC: writing—original draft preparation. AC and VB: writing—review and editing, visualization. VB: supervision and project administration. All authors contributed to the article and approved the submitted version.

## Conflict of Interest

The authors declare that the research was conducted in the absence of any commercial or financial relationships that could be construed as a potential conflict of interest.
